# A simple label-free method reveals bacterial growth dynamics and antibiotic action in real-time

**DOI:** 10.1038/s41598-022-22671-6

**Published:** 2022-11-12

**Authors:** Robert J. H. Hammond, Kerry Falconer, Thomas Powell, Ruth Bowness, Stephen H. Gillespie

**Affiliations:** 1 Division of Infection and Global Health, School of Medicine, University of St Andrews, Oxford, UK; 2 Department of Mathematical Sciences, Centre for Mathematical Biology, University of Bath, Bath, UK

**Keywords:** Preclinical research, Translational research, Biotechnology, Microbiology

## Abstract

Understanding the response of bacteria to environmental stress is hampered by the relative insensitivity of methods to detect growth. This means studies of antibiotic resistance and other physiological methods often take 24 h or longer. We developed and tested a scattered light and detection system (SLIC) to address this challenge, establishing the limit of detection, and time to positive detection of the growth of small inocula. We compared the light-scattering of bacteria grown in varying high and low nutrient liquid medium and the growth dynamics of two closely related organisms. Scattering data was modelled using Gompertz and Broken Stick equations. Bacteria were also exposed meropenem, gentamicin and cefoxitin at a range of concentrations and light scattering of the liquid culture was captured in real-time. We established the limit of detection for SLIC to be between 10 and 100 cfu mL^−1^ in a volume of 1–2 mL. Quantitative measurement of the different nutrient effects on bacteria were obtained in less than four hours and it was possible to distinguish differences in the growth dynamics of *Klebsiella* *pneumoniae* 1705 possessing the *Bla*_*KPC*_ betalactamase vs. strain 1706 very rapidly. There was a dose dependent difference in the speed of action of each antibiotic tested at supra-MIC concentrations. The lethal effect of gentamicin and lytic effect of meropenem, and slow bactericidal effect of cefoxitin were demonstrated in real time. Significantly, strains that were sensitive to antibiotics could be identified in seconds. This research demonstrates the critical importance of improving the sensitivity of bacterial detection. This results in more rapid assessment of susceptibility and the ability to capture a wealth of data on the growth dynamics of bacteria. The rapid rate at which killing occurs at supra-MIC concentrations, an important finding that needs to be incorporated into pharmacokinetic and pharmacodynamic models. Importantly, enhanced sensitivity of bacterial detection opens the possibility of susceptibility results being reportable clinically in a few minutes, as we have demonstrated.

## Introduction

The ability to detect growth is the single most important factor underpinning our understanding of infectious diseases since Pasteur enunciated the “germ theory” of disease and Robert Koch discover new pathogens such as *B.* *anthracis,* and *M.* *tuberculosis*. Our ability to detect inhibition of bacterial growth is the foundation antibiotic discovery from the time of Ehrlich to now. Cultivation of pathogens remains central to microbiological diagnosis. The detection of resistance in sepsis is paramount as there is a 7.6% mean decrease in survival for every hour effective antimicrobial treatment is delayed from its onset^1^. The central paradox is that for organisms that divide every 20 min laboratories often need 12–24 h to detect them. Bacteria can be detected and enumerated on solid medium or by changes in a characteristic of liquid medium. Commonly, this has been achieved by monitoring the pH, carbon dioxide production or changing electrical impedence^2–4^. Many of these techniques can generate a result in as little as four hours in routine practice, but to make further progress we need methods with a lower limit of detection to allow us to monitor bacterial growth in real-time.

Determining the speed of bacterial growth is essential to understand antibiotic action and emergence of resistance. Acquisition of a mobile genetic element or point mutation can affect the physiological fitness of an organism^5^ and this influences the spectrum of mutations. Since Lenski’s seminal experiments, accurate measurement of growth velocity has been used as a proxy for fitness^6,7^. Characterising these changes has a profound impact on our understanding of the likelihood of survival of resistant organisms. Improving these methods would assist our ability to predict the likelihood of a resistant strain surviving and becoming established.

Progress has been made in detecting micro-organisms for diagnosis by non-culture methods. Virus diagnosis by molecular methods is now well established and its application in bacteriology is increasing but the need for susceptibility results limits their scope. Molecular diagnosis requires a comprehensive knowledge of pathogen genetic space, effective design of primers and production of an assay in a user-friendly format. False susceptibility can be reported if the resistance mechanism has not been described and incorporated into diagnostic algorithms. Continuous evolution of microorganisms has resulted in false negative diagnoses when evolution of the pathogen or of a resistance gene means that critical primers no longer bind^8^.

**Figure 1:**
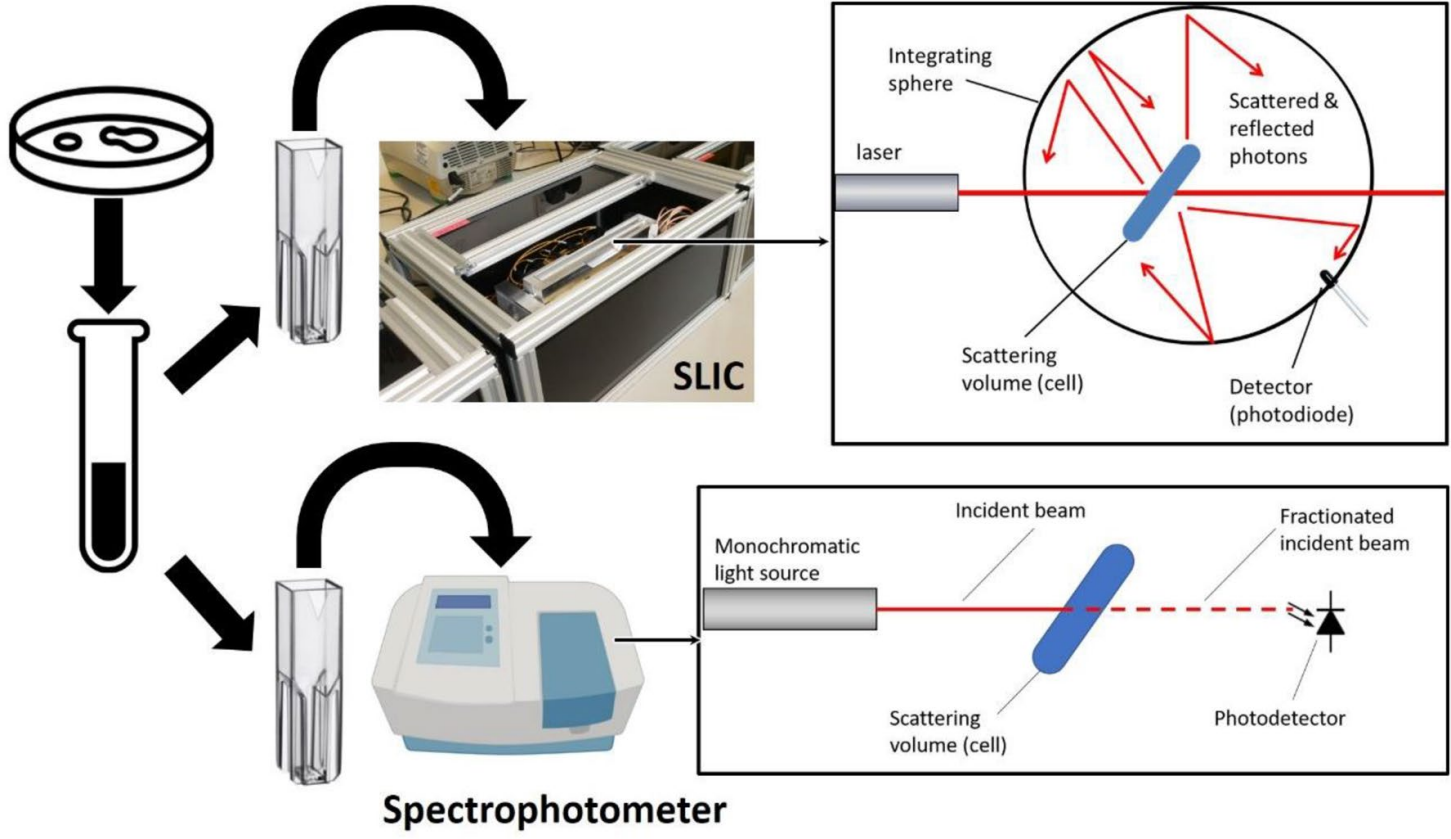
Brief schematic of the laboratory workflow common to both the use of a spectrophotometer and to the most basic use of SLIC, incorporating a diagrammatic representation of the differing light paths in both technologies. Data derived from a comparison experiment akin to the one shown in this figure can be found in Fig. 2.

The aim of our work was to utilise the performance of a new tool to determine whether it was capable of study the growth of bacteria in real-time. This patented technology uses an innovative combination of laser light scattering, locked signal and integrating detection space to achieve this (Patents US10677727B2, US10670521B2, US20190277759A1) (see Fig. 1). The methodology, named scattered light integrated collection (SLIC) provides a very sensitive way to detect microorganisms at low concentrations allowing us to follow their growth in real-time and to study the impact of different stresses on their growth dynamics. A brief summary of how the technology works is as follows. Where a spectrophotometer measures the amount of light absorbed by a sample suspended in liquid medium SLIC does the opposite. SLIC measures the total scattering output from a given scattering volume and disregards the absorbed light (see Fig. 1). This major difference along with the use of a phase locked signal gives SLIC its exquisite sensitivity. In these experiments we use a version of SLIC (SLIC v7) that can detect scatter in multiple samples.

## Results

### Finding the limit of detection

**Figure 2:**
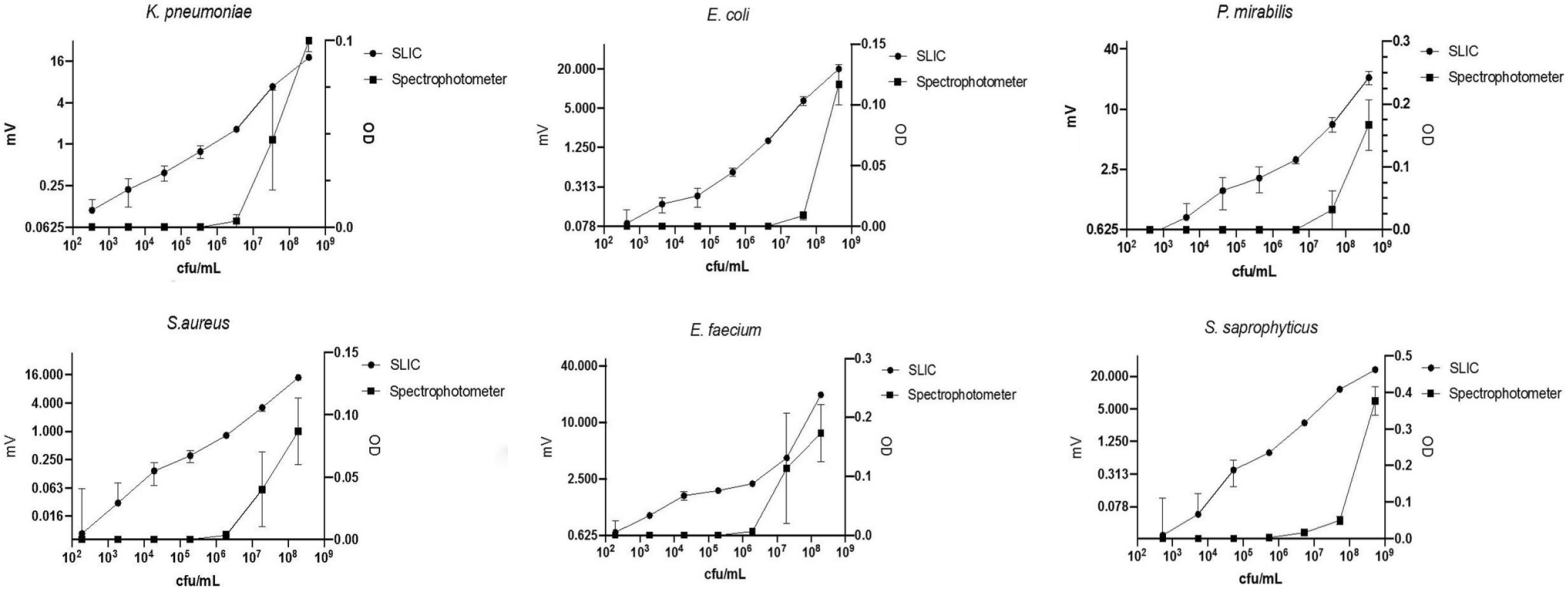
Bacteria of each species were resuscitated from glycerol stock and inoculated into Mueller–Hinton cation adjusted (MH+) media (Sigma) and allowed to incubate overnight (~ 16 h). Seven serial 1:10 dilutions with 100 µL of culture in 900 µL of MH+ media in sterile microcentrifuge tubes (Axel, Germany) were carried out. Each serial dilution was aliquoted into sterile cuvettes, capped and measured in a spectrophotometer (Biochrom WPA CO8000, UK) using sterile MH+ media as a blank. All experiments were performed in triplicate to provide the sensitivity of the spectrophotometer. The same samples were transferred to the SLIC v7 device and scattering signal acquired over 10 s. The average of the three data points were collected. The cuvettes were removed from the SLIC device and for each 3 × 10 µL was aliquoted onto a MH+ agar plate and the number of viable organisms determined by a modified Miles and Misra^7^. Mean values for both parameters were plotted together with the standard error of the mean.

Spectroscopy (OD_600_) is widely used as a standard method to detect and quantify the number of microorganisms rapidly. Thus, we compared the ability of SLIC and spectroscopy to detect the presence of bacteria from the urinary tract commonly isolated in clinical practice. As expected, the limit of detection of bacteria by optical density measurement by spectroscopy was between 10^5^ and 10^7^ cfu ml^−1^ (see Fig. 2). In comparison the level of detection of bacteria using SLIC is as low as 10^2^ cfu mL^−1.^.

SLIC appears to be much more sensitive than spectrophotometry for all bacterial species tested whether, Gram positive or negative, bacilli or cocci. Importantly, the measured response is linear over all of the range of dilutions tested and is still significantly above the baseline even at 100 cfu mL^−1^. This contrasts with optical density measurements where the limit of detection near 10^5^ cfu mL^−1^.

### Time to growth detection

**Figure 3:**
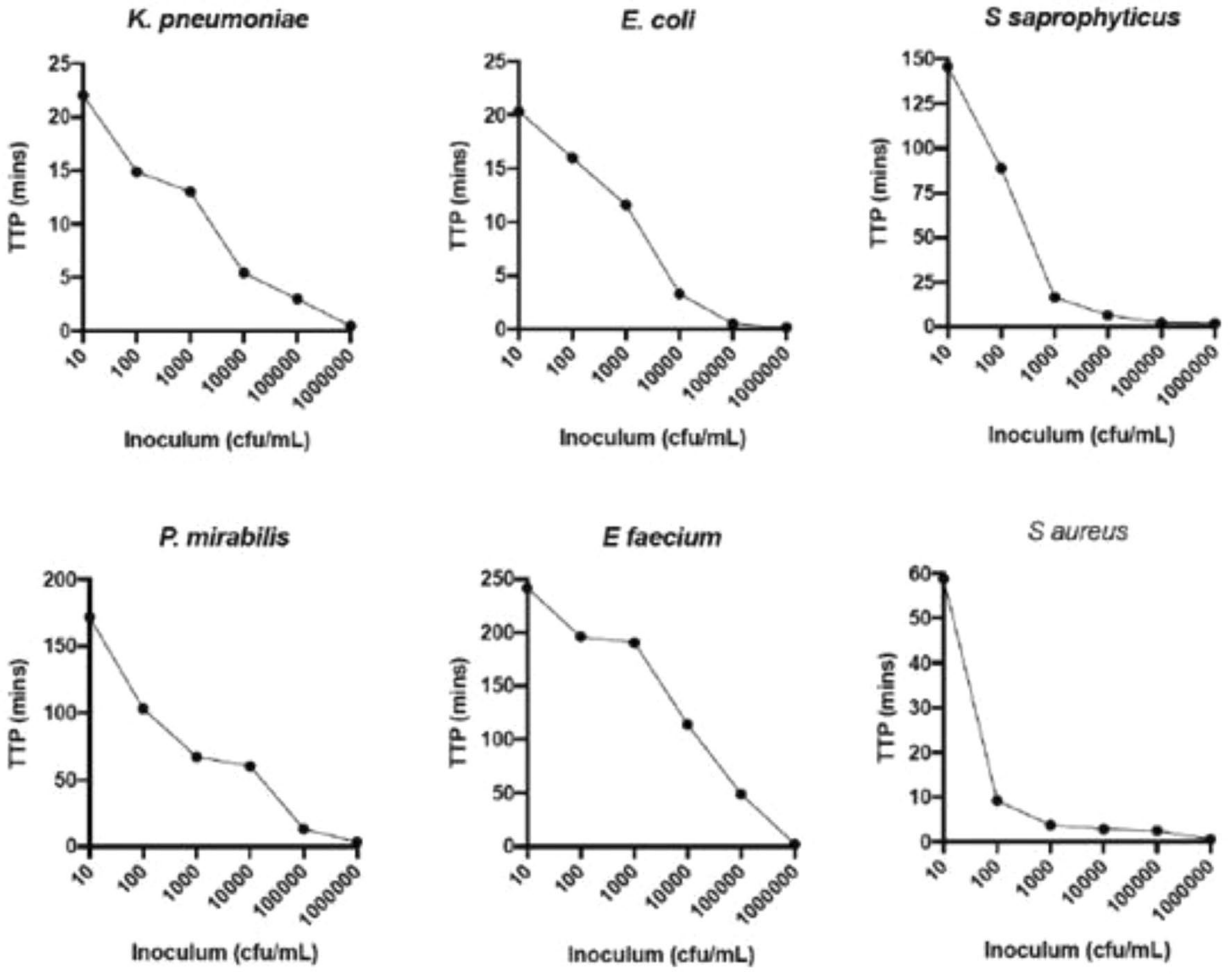
Growing cultures of *K.* *pneumoniae,* *P.* *mirabilis,* *E.* *coli,* *E.* *faecium,* *S.* *saprophyticus* and *S.* *aureus,* were diluted in medium at 37 °C to achieve concentrations between 10^1^ and 10^7^ cfu mL^−1^ and incubated in SLIC for up to 4 h. Time to positivity (TTP) was defined as the point when the mean value for scattering (mV) and 95% confidence intervals were greater than, and remained above, 0 mV (X- axis). n = 3.

It is recognised that methods used to establish the limit of detection can give different results depending on the method of detection, the growth rate of the organisms or the mechanism of division. Thus, we compared the sensitivity of SLIC using the time taken to detect a positive result in liquid medium. A culture of a bacterial species was diluted in medium to give an expected range of concentrations from 10 to 10^6^ cfu mL^−1^ and subsequently confirmed by colony counting^7^. The cultures were incubated in SLIC for up to 4 h and the scattering measured as illustrated in Fig. 3. When the initial inoculum was between 10^4^ and 10^5^ cfu mL^−1^, the concentration commonly used in susceptibility testing, growth was detected in under 15 min (*Enterococcus* *faecium* 6.3 min, *Staphylococcus* *saprophyticus* 2.9 min, *Staphylococcus* *aureus* 3.6 min (Gram positive bacteria), *Klebsiella* *pneumoniae* 0.3 min, *Escherichia* *coli* 1.2 min and *Proteus* *mirabilis* 1.3 min (Gram negative bacteria) respectively). Even at much lower concentrations, for example approximately 100 cfu mL^−1^, three species of Gram-negative organisms, *E.* *coli,* *K.* *pneumoniae* and *P.* *mirabilis*, significant growth could be detected in between 15 and 103 min and a little slower for the three Gram positive species, excepting *S.* *aureus* (TTP at 100 cfu mL^−1^ 9.13 min) (see Fig. 3).

### Differentiating growth rate

One of the challenges in understanding bacterial responses to changes in the environment is the difficulty in distinguishing small changes in growth in samples due to the relative insensitivity of current growth detection methods. In this context, SLIC should have significant advantage over other methods as the low limit of detection means that small differences in the number of bacteria can be detected. We tested this by exploring three different experimental paradigms: measuring growth in varying nutrient concentrations, comparing the fitness of two closely related organisms and growth in the presence of different concentrations of antibiotic.

**Figure 4:**
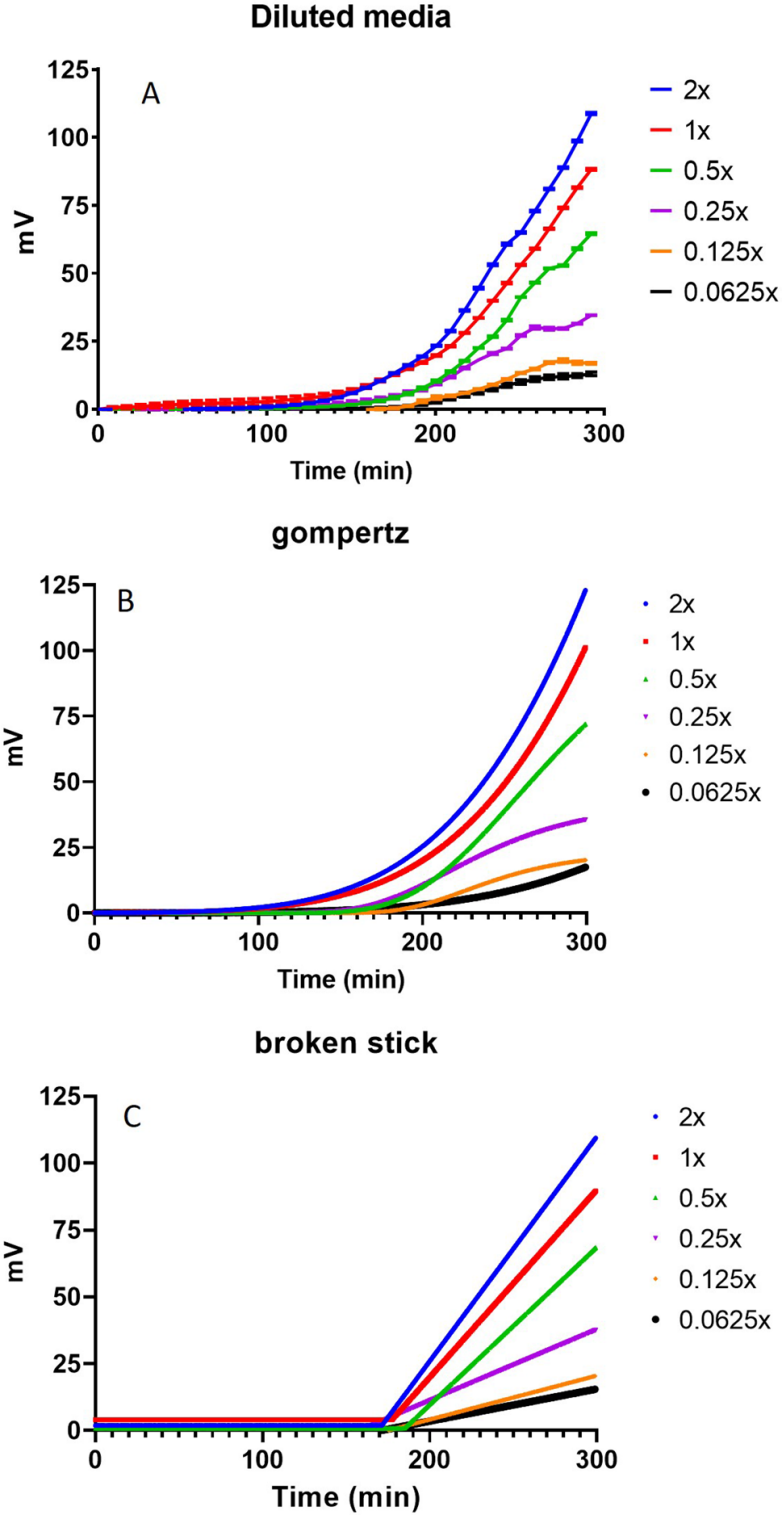
*E.* *coli* ATCC 25922 was diluted to give starting inocula of between 10^1^ and 10^7^ cfu mL^−1^ and cultured in Mueller Hinton cation adjusted broth at 2×–0.0625× the standard concentration. These cultures were incubated for up to 5 h at 37 °C. The points represent the mean of three independent biological observations of output mV. In (**A**), mean data points are linked, and in (**B**), data are fitted to a modified Gompertz equations (solid line) and (**C**). Data are fitted to a Broken Stick equations (hatched line).

To address the question of whether SLIC was able to detect small differences in the number of growing bacteria, we varied the concentration of Mueller Hinton Cation adjusted medium in a range from 2× to 0.0625× and a starting inoculum of *E.* *coli* 25922 at 3.6 × 10^5^ cfu mL^−1^. At high nutrient concentration growth is detected immediately and rises rapidly to achieve a greater degree of scattering. Detection of growth is delayed at lower nutrient concentrations and in this experiment, we show differences across the range of medium concentrations tested (see Fig. 4).

### Growth in medium with varying nutrient concentrations

This shows that bacteria growing at the standard concentration suggested by the manufacturer attained the highest scattering and growth rate. Yet there were only very minor differences in the calculated measure of lag phase, Lambda, as set out in Table 1. Thus, differences in some standard markers of the growth dynamics of an *E.* *coli* strain are readily discernible using SLIC within two hours.

**Table 1:** Values of lag phase ($\lambda$), growth rate ($\mu$), and ${c}_{0}$ for *E.* *coli* ATCC 25922 when fitted to the Broken Stick equation (Eq. 2).

Medium concentration	$\lambda$	$\mu$	${c}_{0}$	AIC
2×	171.44	0.84	1.94	327,305.7
1×	177.46	0.70	4.06	310,119.3
0.5×	184.71	0.59	0.89	258,819.2
0.25×	161.25	0.27	0.89	247,051.8
0.125×	172.18	0.16	− 0.46	195,784.9
0.0625×	169.40	0.12	− 0.15	216,991.3

The organisms were inoculated into Mueller Hinton cation adjusted broth and incubated at 37 °C in SLIC for up to 5 h. Note that these data were also fitted to the Gompertz equation (Eq. 1) but the Broken Stick equation gave better fittings, as determined by lower values for the Akaike Information Criterion (AIC).

### Differentiating microorganisms with different fitness

**Figure 5:**
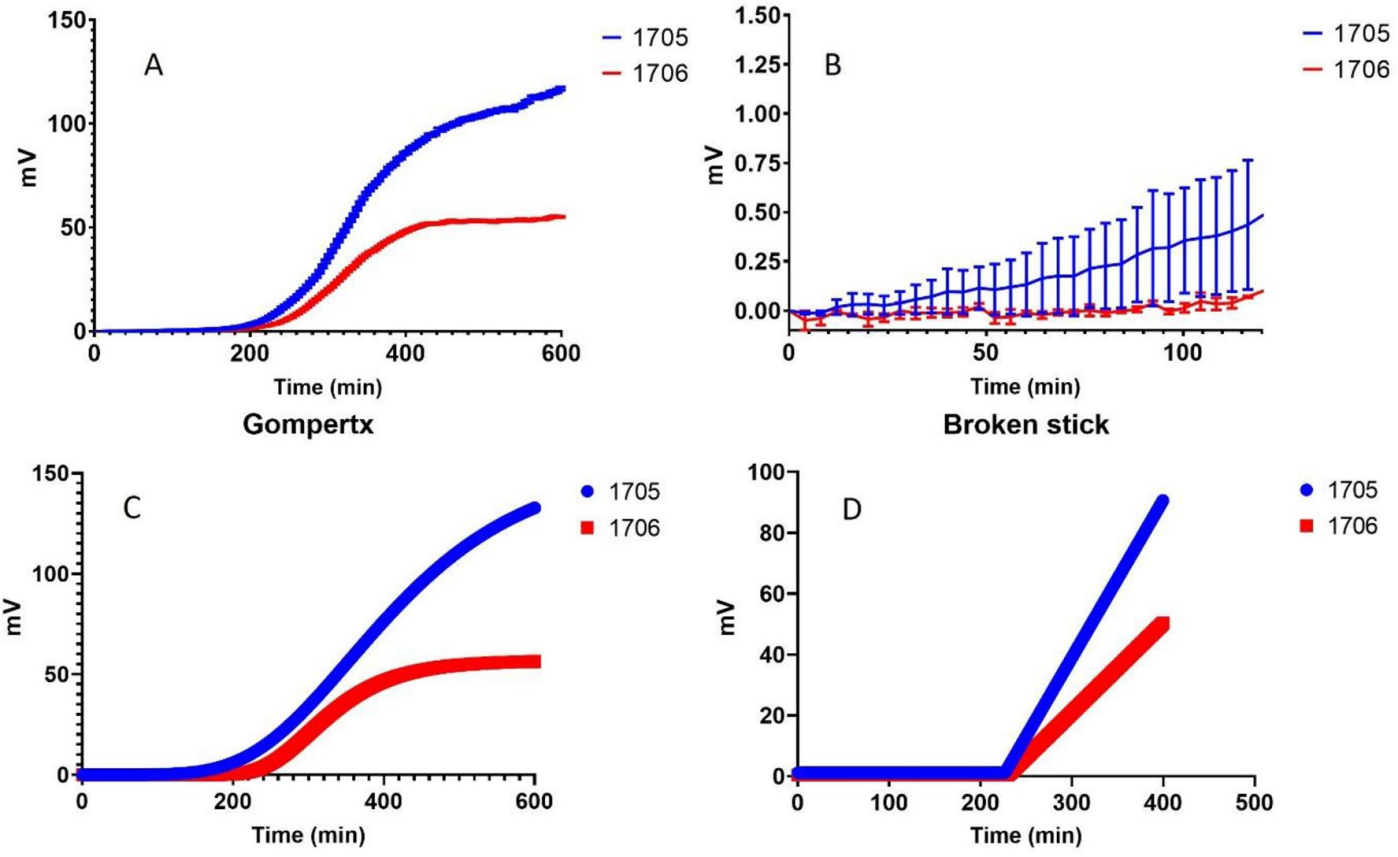
*K.* *pneumoniae* ATCC BAA-1705 and BAA-1706 were cultivated in paralell in SLIC. The output mV data from SLIC v7 over 600 min is plotted in (**A**) with the data for the first 120 min of the experiment is shown in (**B**) (one measurement per second with one in 480 points included in the graph). Points represent the mean of three independent measurements and error bars are the standard error of the mean. All data were fitted to the Gompertz and Broken stick equations (Eqs. 1 and 2, respectively). The fitting results are illustrated in (**C,D**). Note from Table 2 that the Broken Stick gave better fittings than the Gompertz equation, as determined by lower AIC values (see Table 2).

SLIC was used to distinguish growth rate differences in two closely related *Klebsiella* *pneumoniae* that differ by the possession of a plasmid as illustrated in Fig. 5 with differences in calculated growth parameters in Table 2. *Klebsiella* *pneumoniae* 1705 possesses a *bla*_*KPC*_ betalactamase that strain 1706 lacks and is resistant to carbapenems due to the presence of an Amp-C type beta-lactamase enzyme^9,10^.

**Table 2:** Values of lag phase ($\lambda$), growth rate ($\mu$), asymptote (A) and AIC when fitted to the modified Gompertz equation (Eq. 1), and lag phase ($\lambda$), growth rate ($\mu$), ${c}_{0}$ and AIC when fitted to the Broken Stick equation (Eq. 2) for *Klebsiella* *pneumoniae* ATCC BAA-1705 and BAA-1706.

Strain	$\lambda$	$\mu$	${c}_{0}$	AIC
**Fitted Broken stick equation**				
1705	226.74	0.52	1.16	472,021.3
1706	230.26	0.29	0.45	30,813.9

Strain	$\lambda$	$\mu$	A	AIC
**Fitted to modified Gompertz equation**				
1705	223.07	0.43	154.72	814,537.8
1706	239.89	0.34	56.73	596,875.5

The organisms were inoculated into Mueller Hinton cation adjusted broth and incubated at 37 °C in SLIC for up to 5 h.

### Measuring antibiotic effects using SLIC

We studied the effect of three antibiotics with different modes of action on bacterial growth in real-time. The antibiotics selected were meropenem, gentamicin and cefoxitin. These antibiotics were selected due to their different modes of action, the frequency of prescription and due to the observable effect they have on the cells in SLIC.

Meropenem is a bactericidal carbapenem and therefore a β-lactam that works by inhibiting cell wall synthesis by binding penicillin binding proteins (PBPs). Unbound PBPs polymerise peptidoglycan, strengthening the bacterial cell wall. Antibiotics binding PBPs interrupt the process rendering the cell wall fragile or incomplete. Gentamicin is a bactericidal aminoglycoside that interferes with bacterial protein synthesis by binding to the 30S ribosomal subunit. Cefoxitin is another bactericidal β-lactam but a cephalosporin. It similarly works by inhibiting cell wall synthesis by binding PBPs.

**Figure 6:**
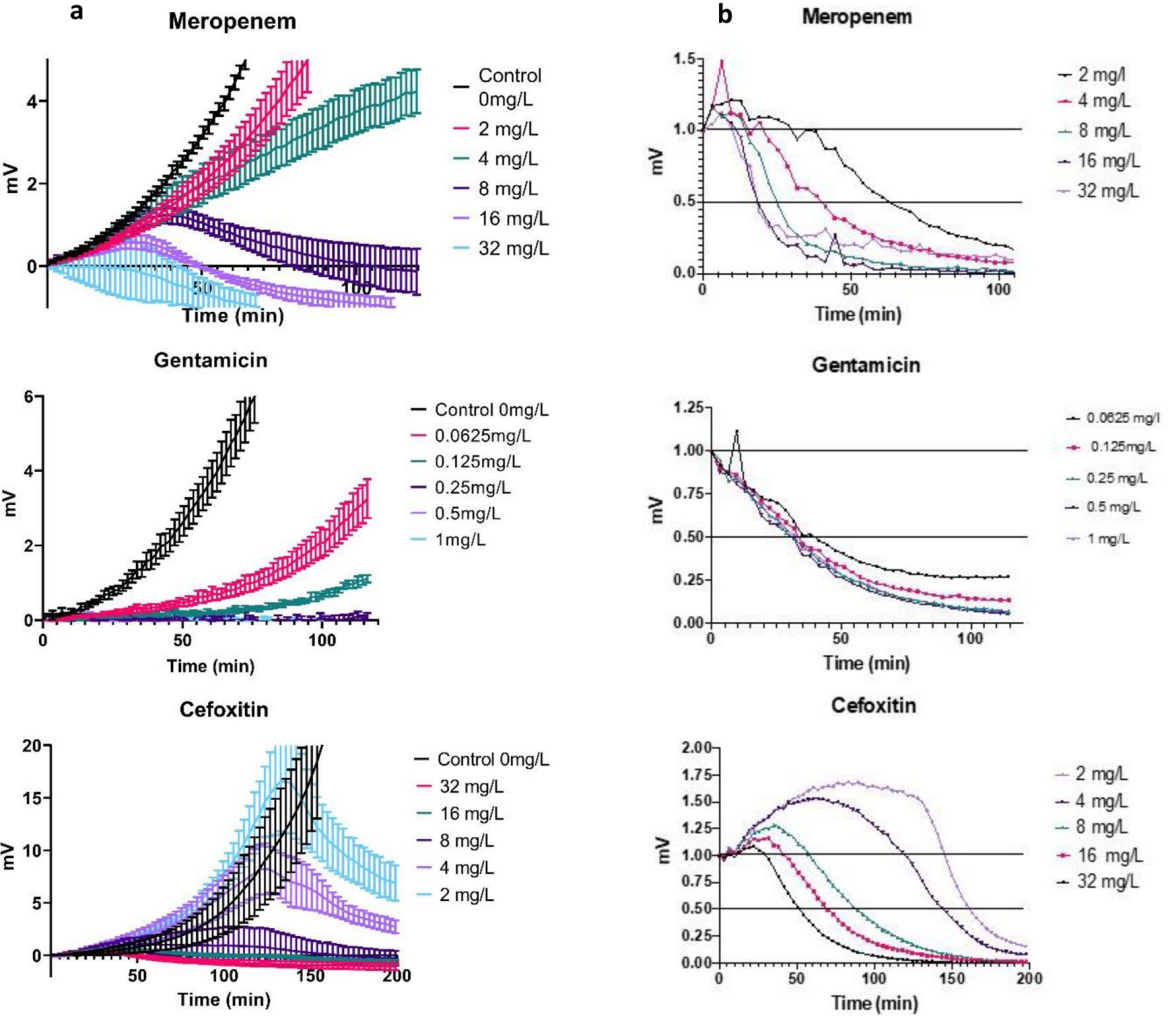
(**a**) An exponentially growing culture of *E.* *coli* ATCC 25922 was cultivated at 37 °C in SLIC in the presence of meropenem (0–32 mg/L), gentamicin (0–1 mg/L) and cefoxitin (0–32 mg/L) in SLIC at 37 °C. Light scattering was detected and plotted in mV against time. Values represent mean and ± 2 standard error. For clarity every 100th data point is plotted. EUCAST MIC for *Enterobacteriaceae* to these agents is as follows: Meropenem—2-8 mg/L. Gentamicin—2 mg/L, Cefoxitin—8 mg/L. One measurement was taken every second. (**b**) This represents the percentage inhibition for the organisms reported in (**a**) but recalculated using the equation (x + 1)/(y + 1) × 100.

When *E.* *coli* was exposed to no antibiotic or increasing concentrations of meropenem the amount of scattering detected in the cultures exposed to antibiotic deviates from the control line very rapidly. This occurs earlier as the concentration of antibiotic increases. It appears almost immediately at 32 mg/L then at 12, 14, 20 and 38 min with each doubling dilution. Intriguingly, all but the 2 and 4 mg/L concentration caused a fall in the detected scattering (see Fig. 6a). A fall in scattering (mV) can only occur in SLIC if cell lysis is occurring. In the case of a bacteriostatic antibiotic the trace would be ‘flat’ (parallel to the x-axis). In the case of a bactericidal antibiotic the trace *may* be flat if the drug is not lytic, or its actions do not cause the cell to lyse through other mechanisms. In the case of a lytic antibiotic the trace will always decline, although not necessarily below the x-axis.

We hypothesise that the decline in signal observed for 8–32 mg/L of meropenem could be due to activation of bacterial autolysins and consequent destruction of cell wall integrity. It could also conceivably be due to a weakening of the cell wall that led to mechanical stress and osmotic imbalance the caused the cell to lyse. This process takes time and accounts for the short plateau seen in the traces for 8–32 mg/L of meropenem (see Fig. 6a) before the signal decline.

We confirmed cellular disruption at 120 min of meropenem exposure (8 mg/L) by performing Gram staining on the material, which shows that meropenem has disrupted the cellular structure of the bacteria, reducing the amount of scattering detected. This time point and concentration of meropenem were chosen as the data indicated that at 120 min cell lysis had occurred, but the trace had only just reached the x-axis therefore the cells must be mostly intact. This is indeed what we found (supplementary Fig. 1).

The amount of scattering in the cultures exposed to varying concentrations of gentamicin also deviated from the no-antibiotic control in a dose dependent fashion. In contrast to the behaviour of meropenem, we found no evidence of cell lysis in any of the time points where the scattering had reached a plateau. We confirmed this result by sampling the latter time points to indicate whether we could detect any intact organisms (data not shown). Scattering by cultures exposed to increasing concentrations of cefoxitin we see a similar relationship between fold difference above MIC and time to divergence from the control line (see Fig. 6b). Yet, in contrast to both other examples, there is a paradoxical increase in scattering. We detected elongated bacterial forms as can be seen in Supplementary Fig. 2. The effect of elongated bacteria on detected scattering was even more pronounced when we calculated the percentage reduction in scattering as demonstrated in Fig. 6.

## Discussion

The study of bacteriology is compromised by the paradox that detection of growth is difficult despite the rapid rate of bacterial cell division. This is due to the relative insensitivity of available detection systems. Here we have shown that SLIC has a limit of detection in the same range as molecular methods such as GeneXpert and TB Molecular bacterial load assay^11,12^. It is much more sensitive than many other culture techniques that require 10^5^ cfu mL^−1^ or automated blood culture before they signal positive at approximately 10^8^ cfu mL^−1^^13^.

The low limit of detection means we could identify small differences in scattering that allow small differences in growth characteristics to be detected. Unlike molecular assays, data emerging from SLIC is dynamic, which means that we can study bacterial behaviour in real time. This was demonstrated by detecting differing growth parameters in the same organism grown in increasingly dilute medium. This property also enabled us to determine the fitness of bacteria and to distinguish the growth dynamics of closely related organisms differing by the presence of a plasmid. The rate of growth is frequently used as a means of measuring the physiological cost of resistance acquisition either by mutation or acquisition of a mobile genetic element^5,6^. This was first identified by Lenski and expanded to include differences in fitness caused by single nucleotide polymorphisms (SNPs) in chromosomal genes that encode resistance. Due to the low sensitivity of culture-based methods, only large differences can be detected. Differentiating fitness deficits of mutants is usually achieved by cultivating a mixture of the parent with the daughter mutant for 24–48 h and plating and counting the proportion of resistant and susceptible colonies^7^. Strains with significant fitness deficits may not be detected due to phenotypic lag^14^. Due to the rapid cell division of bacteria coupled with the exquisite sensitivity of SLIC we are now able to identify small differences in the growth dynamics of organisms in both optimal and sub-optimal conditions. The output that we achieve can be fitted to standard equations of bacterial growth such as the modified Gompertz and the Broken stick method giving measurable parameters^15–18^: duration of lag-phase, growth velocity and maximum cell mass^17,19,20^. Such a development should aid the characterisation of resistant mutants and assist in the understanding of the evolution of resistance and its effect on the physiology of strains that acquire resistance.

The growing threat of antibiotic resistance has prompted the Longitude Prize to set the ambitious target to develop drug susceptibility tests in under 30 min in. In experiments without antibiotics the control line follows an upward curve that would be invisible using standard, less sensitive techniques until much later in the incubation period. The addition of antibiotics can alter this trajectory radically in a dose dependent manner if the test organism is susceptible. One of the most striking observations from our data is that for all three antibiotic classes tested at supra-MIC values there is very little change in the scattering, thus suggesting complete inhibition. The differing growth dynamics of organisms exposed to sub- and supra-MIC amounts of antibiotic is a striking finding with distinct patterns for antibiotics with different modes of action. For meropenem the time taken to deviate from the control we interpret as time taken for the lethal effect of the antibiotic. The subsequent fall in scattering is due to lysis of bacterial cells. A similar pattern is seen with gentamicin but there is no subsequent reduction in scattering which we interpret to be due to the lethal but non-lytic action. With cefoxitin scattering increases initially which we hypothesis is due to inhibition of PBP 1a responsible for cell separation (septal ring closure) and we confirm this explanation by microscopy (supplementary Fig. 2) and non-viability (data not shown). It is also notable that when following these experiments by percentage inhibition the effect of the cephalosporin is delayed relative to both gentamicin and meropenem increasing the time required to provide an accurate assessment of susceptibility.

For all three of the antibiotic classes studied there is a relationship between the multiple above the MIC and the time to deviation from the control. This implies that much higher doses act more quickly. In pharmacodynamics time above MIC or C_max_ are proposed as important markers of antibiotic action and are critical considerations in planning therapy. Our data provides crucial evidence that the multiple above MIC may also be a factor that speeds bacterial killing and needs consideration in future pharmacodynamic experiments.

In a situation where we are anxious to develop rapid tests, this suggests that the determination of a susceptible result could be determined quickly if we had confidence in the sensitivity of the detection method. We propose that the absence of growth in SLIC can be relied on as a marker of susceptibility and determined in seconds. The physician wishes to know whether the strain is susceptible strains whereas most current methods detect resistance: the drugs the patient cannot be treated with. At the standard concentration of organisms used in susceptibility testing (10^5^), numbers of resistant organisms will double in 30 min or less making a change that is easily visible to a system with a low limit of detection. Thus, a system based on SLIC should be able to identify both susceptibility and resistance for most organisms in minutes.

Using a complex microfluidic and time lapse camera system measuring bacterial length in real time Baltekin et al., detected ciprofloxacin action on *E.* *coli* in approximately 15 min^21^. Choi et al., used bacterial imaging and microfluidic systems to measure the area occupied by immobilised microbes^22^. Differing morphological patterns provided susceptibility results in 3–4 h. A biosensor assay using 16S rRNA can detect susceptibility and identify a range of bacteria with a limit of detection of 10^3^ cfu mL^−1^^23,24^. With SLIC we now have a simple low-cost method that can detect antibiotic action as fast as the biological response in the pathogen: we are witnessing direct antibiotic action at a cellular level in real time. This represents the ultimate upper limit on the speed of phenotypic.

There are limitations to the SLIC technology. SLIC exclusively uses liquid phase growth medium, however, as many microbes will grow more readily and rapidly in liquid as opposed to solid media this is not a significant hinderance. Differing growth conditions are of little consequence to SLIC. As a portable device it is possible to place it within a hypoxic chamber if necessary for the growth of oxygen sensitive bacteria. Some bacteria take significantly longer than others to show signs of growth. The use of SLIC with slow growing organisms (mycobacteria, for example) or organisms that readily settle out of the media can be an issue as SLIC has no integrated liquid agitation mechanism. Currently SLIC uses plastic, sterile, capped cuvettes for growth analysis. The use of plastic binding antibiotics such as colistin can be an issue in such circumstances but the inclusion of albumin into the media or the use of glass or quartz cuvettes can similarly defeat these issues.

Our data shows that identifying phenotypic resistance is more challenging. When bacteria are exposed to concentrations near to the MIC it takes an increasing length of time for the growth line to deviate from the control: the measure we have set as a marker of susceptibility. More rapid results could be obtained by additional mathematical processing of the output data. The cephalosporin data shows the time taken to a result is longer meaning that a wide range of antibiotic-species pairs must be tested.

The delay in delivering DST is due to the relative insensitivity of bacterial detection providing an overarching imperative to find low-cost sensitive detection methods. Our focus should change from demonstrating resistance, to demonstrating susceptibility. This has not been possible previously as it was impossible to rely on a “negative” result. With SLIC technology or another with similar sensitivity it is possible to detect susceptibility in minutes providing the physician with the antibiotic that they can treat their patient with but not the drugs they cannot use.

In summary we report the results of experiments with SLIC that provides a sensitive marker of bacterial growth that can also provide quantitative assessment growth dynamics. It shows that antibiotic action occurs very rapidly at significantly supra-MIC concentrations of antibiotics in a dose-dependent fashion. It opens the possibility of susceptibility results being reportable in a few minutes.

## Methods

### Bacteria and culture media

Organisms used in this study were *K.* *pneumoniae* ATCC BAA-1705 and BAA-1706, *E.* *coli* ATCC 25922, *S.* *aureus* ATCC 25923, *S.* *saprophyticus* (patient strain, isolated from Ninewells hospital, Dundee), *E.* *faecium* (patient strain, isolated from Ninewells hospital, Dundee) and *P.* *mirabilis* ATCC 35659. These organisms were grown in Mueller–Hinton Cation Adjusted Broth (MH+) (Sigma-Aldrich).

Organisms were resuscitated from glycerol stocks stored at −80 °C. A loopful of frozen material was inoculated into 10 mL MH+ and incubated overnight (~ 16 h) at 37 °C.

### Finding the limit of detection

Bacteria (*E.* *coli,* *K.* *pneumoniae,* *P.* *mirabilis,* *S.* *aureus,* *S.* *saprophyticus,* *E* *faecium*) were diluted from an initial concentration of approximately 10^9^ cfu mL^−1^ and examined by both SLIC and spectroscopy. Seven serial (10×) dilutions of overnight culture were made into MH+ broth in microcentrifuge tubes. These cultures were transferred to sterile cuvettes (Brand, Germany), capped and measured in a spectrophotometer (Biochrom, WPA CO8000) using sterile MH+ as a blank. The same samples were transferred to the SLIC v7 device and scattering signal acquired over 10 s. Experiments were run in triplicate and data collected every 20 ms (50 Hz) and averaged. Cuvettes were removed from the SLIC device and 3 × 10 µL was inoculated onto Mueller–Hinton Cation adjusted agar medium (Oxoid). The viable count was determined by a modified Miles and Misra as described previously^7^.

We compared the sensitivity of SLIC using the time taken to detect a positive result in liquid medium and defined a positive result as a signal that was stable or rising. Statistical tests were used to establish significance from the baseline (x-axis). A stable or rising signal is one that has trended away from the baseline and remains significantly above baseline for the duration of the experiment. Baseline accuracy was established using negative controls (sterile media blanks, data not shown).

### Time to positivity (TTP)

Six serial tenfold dilutions of overnight culture were made into MH+ broth in microcentrifuge tubes. These cultures were transferred to sterile cuvettes, capped and measured in a spectrophotometer using 37 °C sterile MH+ as a blank. All experiments were performed in triplicate. The same samples, warmed to 37 °C, were transferred directly to the SLIC v7 device for measurement. The SLIC device was pre-warmed to 37 °C. SLIC is fitted with a temperature feedback system to control the thermal properties of the internal cavity to within 0.001 °C. The cuvettes, media and SLIC being at 37 °C and the minimal transfer times mean that the heat losses during experimental set up are minimal and do not affect bacterial growth characteristics. Data was acquired every second until sustained growth, defined as sustained deviation from the X-axis, had been detected (up to 4 h).

### Differentiating growth rate

#### Growth in medium with varying nutrient concentrations

Bacterial preparations of *E.* *coli* ATCC 25922 were prepared as follows: six overnight culture aliquots were made into MH+ broth in microcentrifuge tubes. These cultures were transferred to sterile cuvettes, capped and measured in a spectrophotometer using 37 °C sterile MH+ as a blank. Overnight cultures were interrogated in a spectrophotometer (OD_600_) and their starting concentrations normalised by measurement of the optical density. The culture was diluted such that the spectrophotometer reading would be < 0.01 that we had previously standardised as giving a viable count of approximately 5 × 10^6^ cfu mL^−1^. All experiments were performed in triplicate. As before, all samples were warmed to 37 °C, were transferred directly to the SLIC v7 device for measurement.

Varying nutrient content media were prepared by making a total of six doubling dilutions from 2× to 0.0625× normal strength. Cultures were then aliquoted into these various concentrations of warmed media at a final concentration of ~ 5 × 10^5^ cfu mL^−1^ and loaded directly into a pre-warmed (37 °C) SLIC device and recordings taken for up to 5 h. Frequency of observation was 1 measurement per second per cuvette (18,000 measurements in 5 h per cuvette).

#### Differentiating microorganisms with different fitness

Two *K.* *pneumoniae* strains (ATCC BAA 1705 & 1706) were prepared from glycerol stock immediately before the experiment began. The strains were resuscitated from −80 °C into 37 °C MH+ media and incubated for 6 h. The cultures were assessed for confluence by spectrophotometer. Sufficient growth was evaluated to be > 0.1 OD_600_. Cultures were then diluted in 37 °C MH+ media to ~ 5 × 10^6^ cfu mL^−1^ (as described above) and loaded into SLIC at a final concentration of ~ 5 × 10^5^ cfu mL^−1^ and scattering measured immediately and continued for up to 10 h. Frequency of observation was 1 measurement per second per cuvette (36,000 measurements in 10 h per cuvette).

### Measuring antibiotic effects using SLIC

#### Preparation of antibiotics

Gentamicin, meropenem and cefoxitin (Sigma-Aldrich, UK) stock solutions were prepared as described previously. The working concentrations were meropenem—320, 160, 80, 40, 10 mg mL^−1^, gentamicin—10, 5, 2.5, 1.25, 0.625 mg mL^−1^, cefoxitin—320, 160, 80, 40, 10 mg mL^−1^. These solutions were aliquoted into the SLIC cuvettes first, followed by the 37 °C MH+ media and finally by the ~ 5 × 10^6^ cfu mL^−1^ bacterial preparations. Temperature loss due to the inclusion of the room temperature drug concentrations was minimal and compensated for using warmed cuvettes and an insulated cuvette rack.

#### Preparation of SLIC cuvettes

A 200 µL of antibiotic solution was added at 10× final concentration to a sterile cuvette. An aliquot of 1600 µL of sterile MH+ was added and 200 µL of diluted culture stock was added (as in “Finding the limit of detection”, final concentration ~ 5 × 10^5^ cfu mL^−1^) to make a final volume of 2 mL. These cuvettes were then loaded into SLIC and scattering measured for up to 200 min. Frequency of observation was 1 measurement per second per cuvette (12,000 measurements in 200 min per cuvette).

#### Calculation of bacterial growth dynamics

We fitted the data to a Gompertz formula^16^ and to an alternative “Broken Stick” formula^18^

Gompertz formula:

1 $$Y=A{e}^{-{e}^{\frac{\mu (\lambda -t)}{A}+1}},$$

Broken Stick formula:

2 $$Y={c}_{0}+\mu \left(t-\lambda \right)\frac{sign\left(t-\lambda \right)+1}{2},$$

where $\lambda$ indicates the lag phase, $\mu$ denotes the growth rate, A indicates the value of the asymptote for the Gompertz equation and ${c}_{0}$ is a measure of shift from zero on the y-axis at time = 0 for the Broken Stick equation.

## Supplementary Information

Supplementary Figures.

## Data Availability

The datasets used and/or analysed during the current study available from the corresponding author on reasonable request. The datasets generated and/or analysed during the current study are not publicly available due to commercial sensitivity and length. Each SLIC run consists of hundreds or thousands of individual data points at it is not practical to upload these to an off-site server unless required. Data are, however, available from the corresponding author on reasonable request.
