# Supplementary material for: A simple label-free method reveals bacterial growth dynamics and antibiotic action in real-time
Source: Sci Rep. 2022 Nov 12;12:19393. doi: 10.1038/s41598-022-22671-6 (PMC9653415; doi:10.1038/s41598-022-22671-6)
Supplement: Supplementary file 1 — Supplementary Figures. [file 41598_2022_22671_MOESM1_ESM.docx]

**Supplementary material**

**
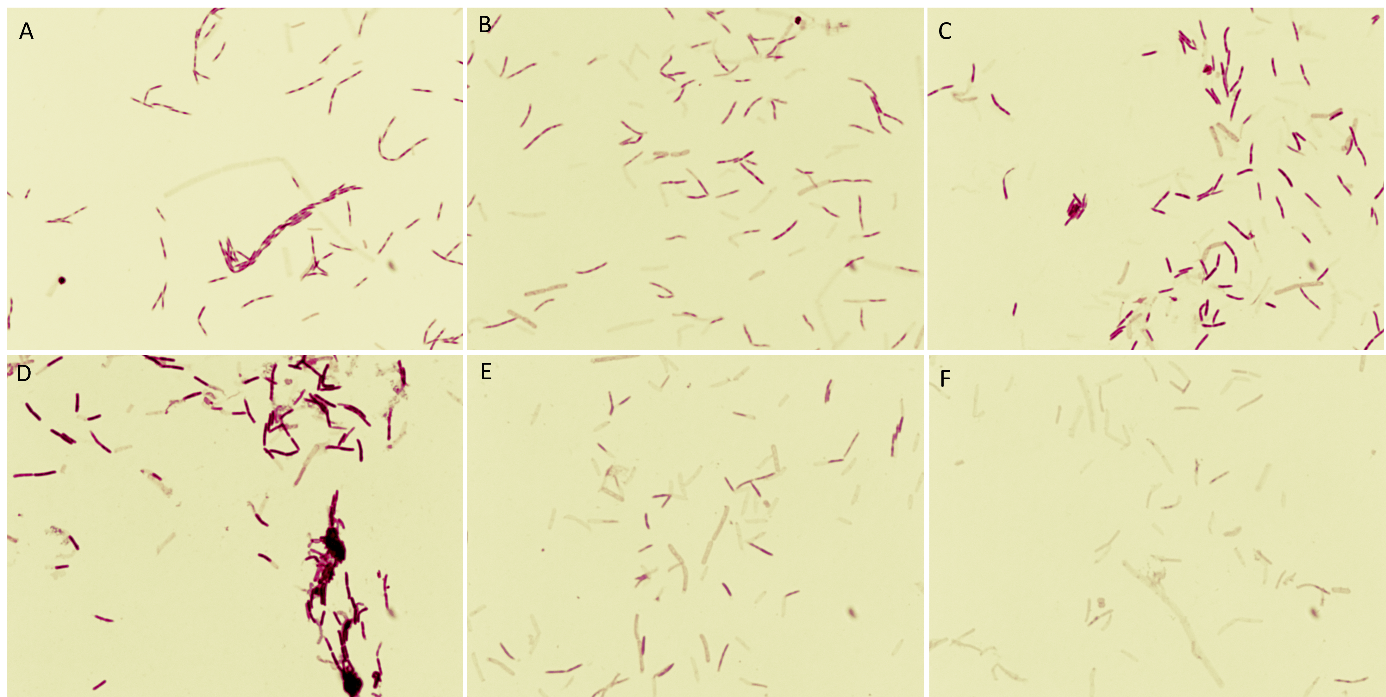
**

**Supplementary figure 1 A-F. *E. coli* ATCC 25922 Gram stained after treatment with meropenem. (0-mg/L, A. 2 mg/L, B. 4 mg/L, C. 8 mg/L, D. 16 mg/L, E. 32 mg/L, F. Sampled at the end of the experiment; 120 minutes. Hazy cell outlines indicate cell wall damage due to antibiotic exposure.**


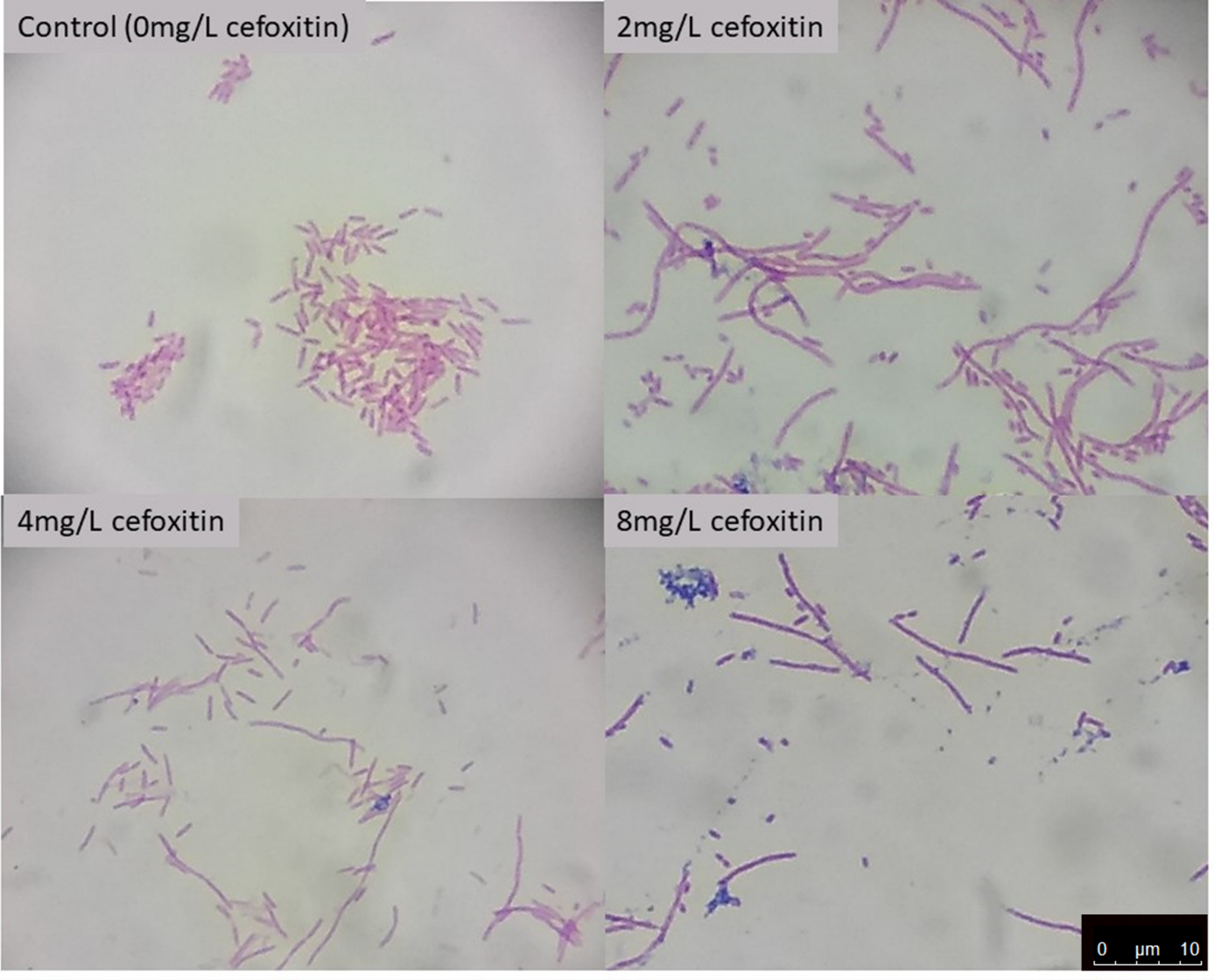
**Supplementary figure 2. *E. coli* ATCC 25922 exposed to cefoxitin at the indicated concentrations, sampled at 50 minutes post exposure (see figures 5a & 5b).**
